# The Selective Advantage of Synonymous Codon Usage Bias in *Salmonella*

**DOI:** 10.1371/journal.pgen.1005926

**Published:** 2016-03-10

**Authors:** Gerrit Brandis, Diarmaid Hughes

**Affiliations:** Department of Medical Biochemistry and Microbiology, Uppsala University, Biomedical Center, Uppsala, Sweden; The Ohio State University, UNITED STATES

## Abstract

The genetic code in mRNA is redundant, with 61 sense codons translated into 20 different amino acids. Individual amino acids are encoded by up to six different codons but within codon families some are used more frequently than others. This phenomenon is referred to as synonymous codon usage bias. The genomes of free-living unicellular organisms such as bacteria have an extreme codon usage bias and the degree of bias differs between genes within the same genome. The strong positive correlation between codon usage bias and gene expression levels in many microorganisms is attributed to selection for translational efficiency. However, this putative selective advantage has never been measured in bacteria and theoretical estimates vary widely. By systematically exchanging optimal codons for synonymous codons in the *tuf* genes we quantified the selective advantage of biased codon usage in highly expressed genes to be in the range 0.2–4.2 x 10^−4^ per codon per generation. These data quantify for the first time the potential for selection on synonymous codon choice to drive genome-wide sequence evolution in bacteria, and in particular to optimize the sequences of highly expressed genes. This quantification may have predictive applications in the design of synthetic genes and for heterologous gene expression in biotechnology.

## Author Summary

The universal genetic code is redundant, with some amino acids being encoded by up to six different codons. Synonymous codons are not used randomly and typically one codon is used more frequently than others. This biased use of codons has been observed in all branches of life. Codon bias is the result of long-term selection and is presumed to confer an evolutionary advantage. The nature of this selective advantage in free-living bacteria, where extreme bias is observed in highly expressed genes, is not fully understood. Furthermore, the magnitude of the fitness benefit of a single optimal codon is so small that it has never been measured experimentally. By systematically exchanging multiple codons at the same time we were able to increase the fitness effects of codon usage to a measurable level. Based on these data we determined the average strength of selection that acts on codon choice in highly expressed genes and show that codon usage bias is selected to optimize the speed of protein production. Quantification of codon usage bias deepens our understanding of the selective forces driving long-term genome evolution and might also find applications in the rational design of synthetic genes.

## Introduction

Synonymous codon usage bias refers to differences in the relative frequency of synonymous codons for individual amino acids in protein coding sequences. Mutation, selection, and random genetic drift are the three major forces that shape codon usage bias in different organisms [1–3]. Fast-growing microorganisms, including *Escherichia coli* and *Salmonella* Typhimurium, have an extreme codon usage bias that correlates with gene expression level [4, 5]. This biased codon usage is thought to be selected for translational efficiency but it is still unclear whether selection is primarily for translation speed or translation accuracy [6–8]. Currently there is no experimental data on the magnitude of the selection for codon bias in highly expressed genes in bacteria, and theoretical approaches have resulted in two very different values being proposed. Based on an analysis of synonymous nucleotide polymorphisms in the *gnd* gene from natural isolates of *E*. *coli* a selection coefficient against non-optimal codons was calculated to be around 10^−9^ per codon per generation [9]. In contrast, another theoretical study arrived at a selection coefficient of approximately 10^−4^ per codon per generation, based on selection-mutation-drift theory [1]. The few experimental studies that have addressed codon usage bias have found correlations between the observed selective disadvantage and the presence of Shine-Dalgarno-like sequences and mRNA folding [10–13] and have associated insertions of very rare codons with increased translational errors, with reading frame shifts, and with reducing the rate of translation [14–16]. These studies do not lead to general conclusions, on either the magnitude, or the selective basis, of synonymous codon usage bias and its association with fast growth rates and highly expressed genes. Here we have experimentally measured the general selective value of synonymous codon bias by substituting non-optimal synonymous codons throughout the coding sequence of two very highly expressed genes, *tufA* and *tufB*. This experimental approach addresses two important questions: (i) the magnitude of selection for optimal codons in highly expressed genes; and (ii) whether synonymous codon usage bias is selected to maximize translation speed or translation accuracy.

## Results

*Salmonella* Typhimurium LT2, a genetically tractable and free-living bacterial species with a strong selection for codon bias [5] was used as the model organism for this study. Because the bias in synonymous codon usage is strongest in highly expressed genes [17, 18] we chose the highly conserved and highly expressed *tuf* genes as the targets for experimentally measuring the selective effects of synonymous codon changes. Two widely separated genes, *tufA* and *tufB*, encode translation elongation factor EF-Tu, the most highly expressed protein in *Salmonella*. In rich media approximately 9% of the protein mass of exponentially growing *Salmonella* is EF-Tu, and bacterial growth rate is strictly correlated to EF-Tu abundance [19].

**Fig 1:**
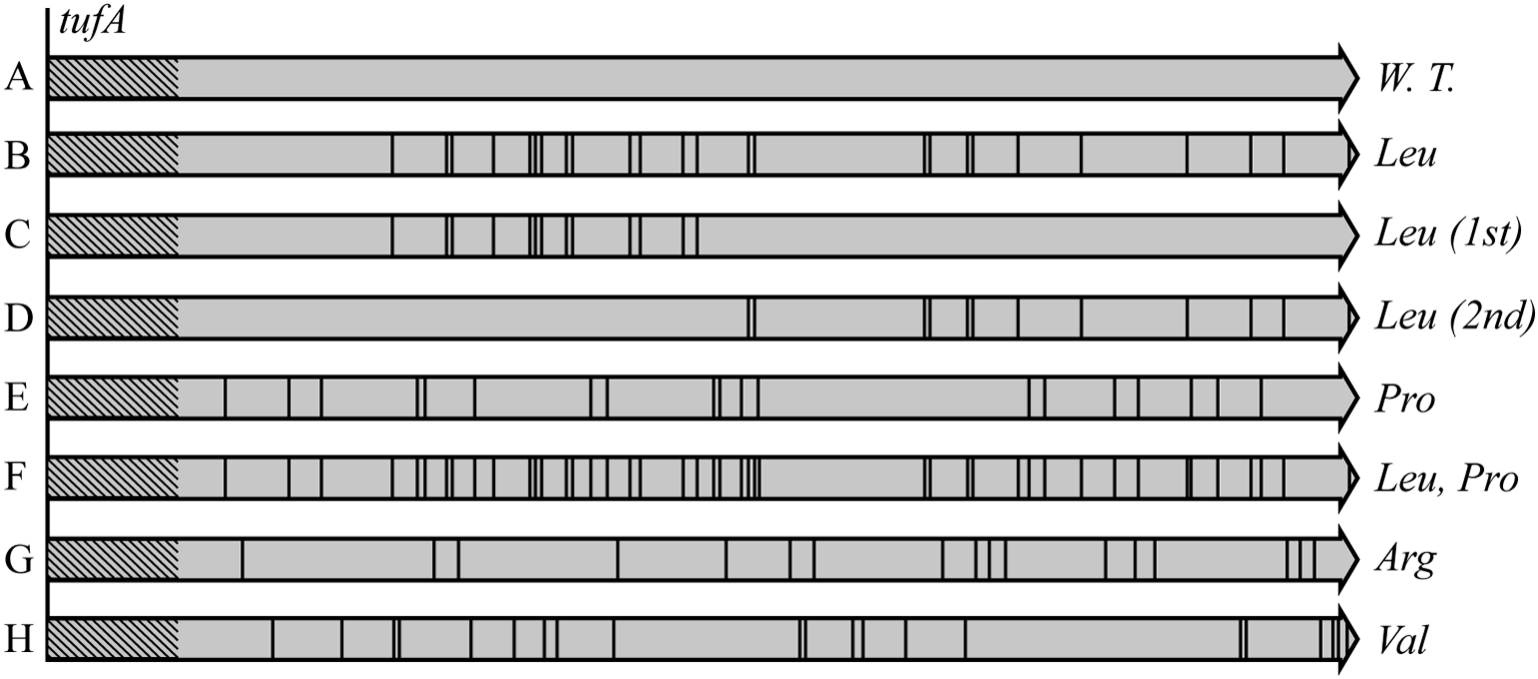
Overview of the design of synonymous *tuf* alleles. The first forty codons in *tuf* were left unchanged to reduce the possible impact of N-terminal codon usage bias (hatched region). Black bars represent codons that have been changed to synonymous codons. (**A**) Wild-type *tufA* gene. (**B**) Leucine codons (N = 25) were changed to UUA, UUG, CUU, CUC or CUA, respectively. (**C**) The leucine codons in the first half of the *tuf* gene (N = 13) were changed to UUA, CUC or CUA, respectively. (**D**) The leucine codons in the second half of the *tuf* gene (N = 12) were changed to UUA, CUC or CUA, respectively. (**E**) Proline codons (N = 19) were changed to CCU, CCC or CCA. (**F**) Both leucine and proline codons (N = 44) were changed: in one *tuf* allele all leucine codons were changed to UUG and all proline codons to CCU; in the other *tuf* allele all leucine codons were changed to UUA and all proline codons to CCA. (**G**) All arginine CGU codons (N = 17) were changed to CGG. (**H**) All valine GUU codons (N = 21) were changed to GUC.

Eighteen different *tuf* alleles were synthesized, in which the optimal codons for one or more of four different amino acids (leucine, proline, valine, and arginine) were systematically replaced with ten different synonymous codons (Fig 1 and S1 Table). In each novel *tuf* gene multiple codons (12 to 25) for a particular amino acid were changed to a less-frequently used synonymous codon for that amino acid. These novel *tuf* genes encode wild-type EF-Tu and each of them supports bacterial viability even when present as the only *tuf* gene in the chromosome. A set of isogenic strains for use in experimental quantification of the selective advantage of codon usage bias, were made by placing each of the eighteen different *tuf* alleles in the chromosome at both native *tufA* and *tufB* positions. The relative fitness cost of each of the synonymous *tuf* alleles was measured using a high-resolution growth competition assay (Materials and Methods). The outcome of each competition assay was used to calculate the average selective disadvantage per non-optimal synonymous codon. The robustness of the selective values based on individual synonymous codons was assessed by also measuring the competitive fitness associated with *tuf* alleles in which only half of the codons were changed to synonymous codons and *tuf* alleles in which codons for two different amino acids were simultaneously changed to their respective synonymous codons.

There was a selective disadvantage associated with having synonymous ‘non-optimal’ codons in the *tuf* genes that ranged between 0.04 and 0.72 x 10^−2^ per generation for the different *tuf* alleles. This is equivalent to an average cost of 0.2 to 4.2 x 10^−4^ per codon per generation (Table 1 and S1 Fig).

**Table 1:** The selective disadvantage of synonymous *tuf* alleles.

Amino acid Codon		N^a^	DDG^b^	N hexamers with anti-SD affinity		Log(*wa*)^c^	*Δt*_*trans*_^d^	*s*^e^ [95% C. I.] x 10^−2^ (total)	*s*^e^ [95% C. I.] x 10^−4^ (per codon)
				*< -4 kcal/mol*	*< -6 kcal/mol*				
Leu	UUA	25	44.0	8	0	-1.53	0.23	0.72 [0.42; 1.02]	2.89 [1.68; 4.10]
Leu	UUA	13	24.2	8	0	-1.53	0.23	0.25 [0.13; 0.38]	1.92 [1.00; 2.85]
Leu	UUA	12	20.7	8	0	-1.53	0.23	0.30 [0.15; 0.45]	2.50 [1.25; 3.87]
Leu	UUG	25	11.3	8	0	-1.45	0.04	0.23 \[0.05; 0.41]	0.92 [0.20; 1.64]
Leu	CUU	25	23.9	8	0	-1.25	0.13	0.58 [0.40; 0.76]	2.33 [1.61; 3.05]
Leu	CUC	25	25.9	8	0	-1.33	0.13	0.59 [0.35; 0.83]	2.37 [1.41; 3.33]
Leu	CUC	13	11.9	8	0	-1.33	0.13	0.28 [0.18; 0.38]	2.15 [1.38; 2.92]
Leu	CUC	12	14.3	8	0	-1.33	0.13	0.31 [0.22; 0.41]	2.58 [1.75; 3.42]
Leu	CUA	25	27.4	8	0	-2.41	0.28	0.63 [0.44; 0.82]	2.53 [1.77; 3.29]
Leu	CUA	13	15.0	8	0	-2.41	0.28	0.26 [0.20; 0.32]	2.00 [1.54; 2.46]
Leu	CUA	12	16.1	8	0	-2.41	0.28	0.29 [0.18; 0.40]	2.42 [1.50; 3.33]
Leu	CUG	0	0	8	0	0	0	0	0
Pro	CCU	19	22.1	7	0	-0.72	-0.03	0.04 [-0.14; 0.22]	0.21 [-0.74; 1.16]
Pro	CCC	19	27.8	14	0	-1.99	0.08	0.29 [0.12; 0.46]	1.53 [0.63; 2.43]
Pro	CCA	18	12.8	7	0	-0.75	0.20	0.27 [0.16; 0.38]	1.50 [0.89; 2.11]
Pro	CCG	0	0	8	0	0	0	0	0
LeuPro	UUGCCU	44	30.2	7	0	-0.99	0.04	0.57 [0.31; 0.73]	1.30 [0.71; 1.89]
LeuPro	UUACCA	43	62.5	7	0	-1.04	0.25	0.70 [0.50; 0.90]	1.63 [1.16; 2.10]
Arg	CGG	17	-25.8	20	3	-2.31	0.40	0.72 [0.53; 0.91]	4.25 [3.13; 5.37]
Arg	CGU	0	0	8	0	0	0	0	0
Val	GUC	21	-7.7	8	0	-0.75	0.10	0.53 [0.37; 0.69]	2.53 [1.76; 3.30]
Val	GUU	0	0	8	0	0,00	0,00	0	0

^a^ ‘N’ is the number of codons that differ between the synonymous and the wild type *tuf* allele.

^b^ ‘DDG’ is the absolute change in predicted mRNA free energy between the synonymous *tuf* alleles and *tufA*.

^c^ ‘log(*wa)*’ is the logarithm of the relative adaptiveness of a codon.

^d^ ‘*Δt*_*trans*_’ is the increase in translation time for the synonymous codon compared to the wild-type codon.

^e^ ‘*s’* is the selective disadvantage per generation shown for the full synonymous *tuf* allele and per synonymous codon.

The calculation of the average fitness cost per codon is based on the assumptions that all synonymous substitutions (a) contribute equally to the total fitness cost and (b) are independent from each other in their effect on fitness.

**Fig 2:**
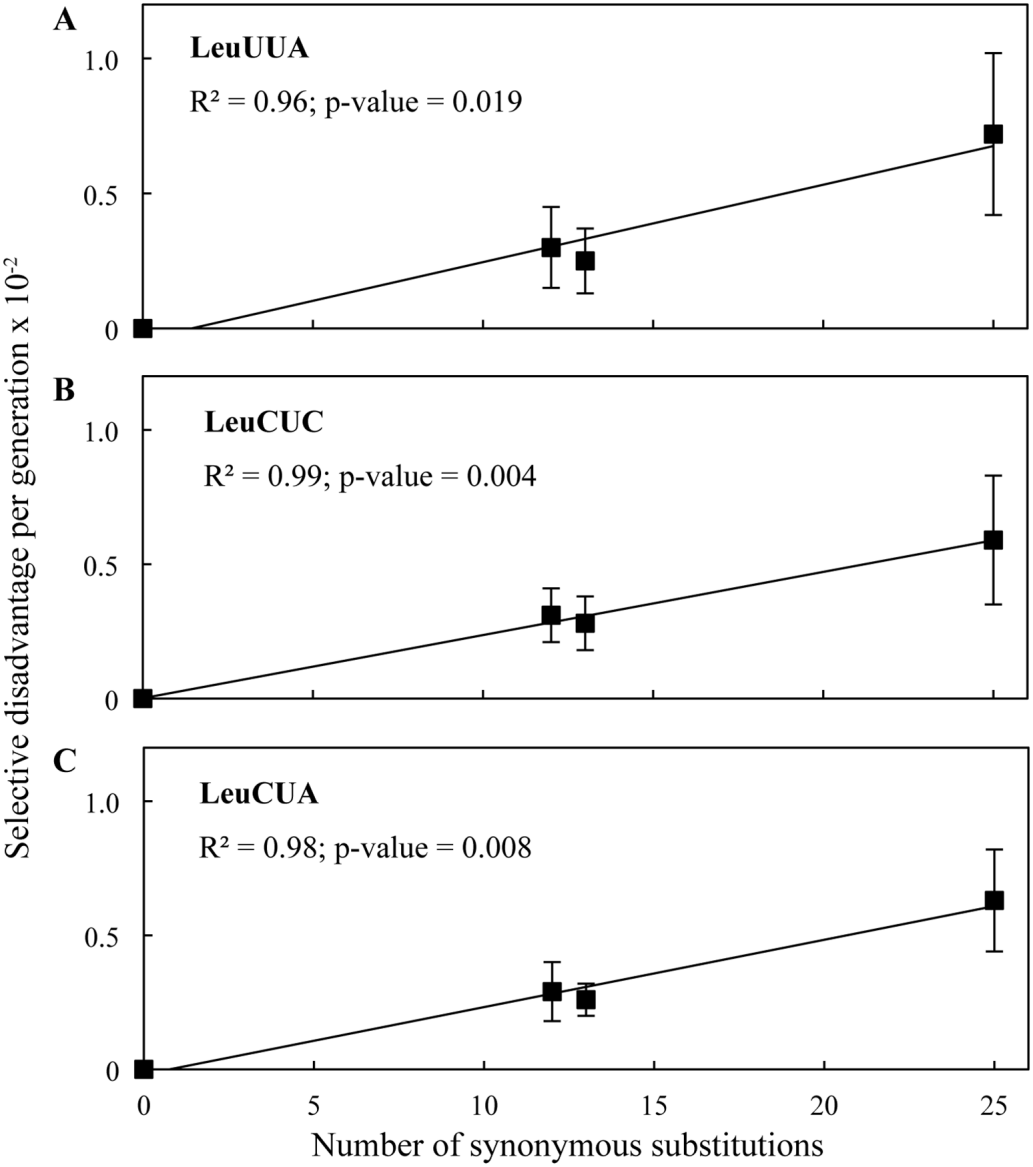
Correlation between the number of synonymous substitutions and the resulting selective disadvantage. Selective disadvantage as a function of the number of synonymous substitutions for the leucine codons **(A)** UUA, **(B)** CUC and **(C)** CUA. Results are shown as means ± 95% confidence interval. Black line indicates regression fit.

Nine of the synonymous *tuf* alleles were constructed to test whether or not the synonymous substitutions contribute equally to the total fitness cost. In these alleles the leucine codons in either the first half, the second half, or all of the tuf gene, were changed to the synonymous codons UUA, CUC or CUA, respectively. If all synonymous substitutions contribute equally then there should be a direct correlation between the number of synonymous substitutions and the total selective disadvantage of the respective *tuf* allele. For all three tested synonymous leucine codons the number of substitutions correlated well with the selective disadvantage (r-squared = 0.96; p-value 0.019 (LeuUUA); r-squared = 0.99; p-value 0.004 (LeuCUC) and r-squared = 0.98; p-value 0.008 (LeuCUA)) (Fig 2). Using these correlations to calculate the selective disadvantage per codon results in 2.87 x 10^−4^ (LeuUUA), 2.35 x 10^−4^ (LeuCUC) and 2.51 x 10^−4^ per codon per generation (LeuCUA), which is indistinguishable from the values determined by substituting all 25 leucine codons. These data are consistent with the assumption that all codons contribute equally to the total selective disadvantage.

If synonymous mutations act independently of each other then it is expected that the combined fitness costs of two *tuf* alleles with distinct synonymous substitutions would be equal to the fitness cost of a single *tuf* allele with the combined substitutions. This is the case for the three pairs of *tuf* alleles in which the leucine codons in either the first or the second half of the *tuf* gene were substituted with synonymous codons. The sum of the selective disadvantages of the two alleles with half the codons changed is not significantly different from the selective disadvantage of the allele in which all of the codons were changed at the same time (p-values 0.47 (LeuUUA); 1.00 (LeuCUC) and 0.56 (LeuCUA)). Two synonymous *tuf* alleles in which the codons for two amino acids, leucine and proline, were both replaced were constructed to test if the assumption of independence is also valid for changes involving multiple amino acids in the same *tuf* allele. For both tested alleles there was no significant difference between the fitness cost of the double mutant allele and the sum of the fitness costs of the two respective single mutant alleles (p-values 0.11 (LeuUUG, ProCCU) and 0.13 (LeuUUA, ProCCA)) indicating that the synonymous mutations act independently to reduce fitness even when codons for multiple amino acids are changed at the same time.

**Fig 3:**
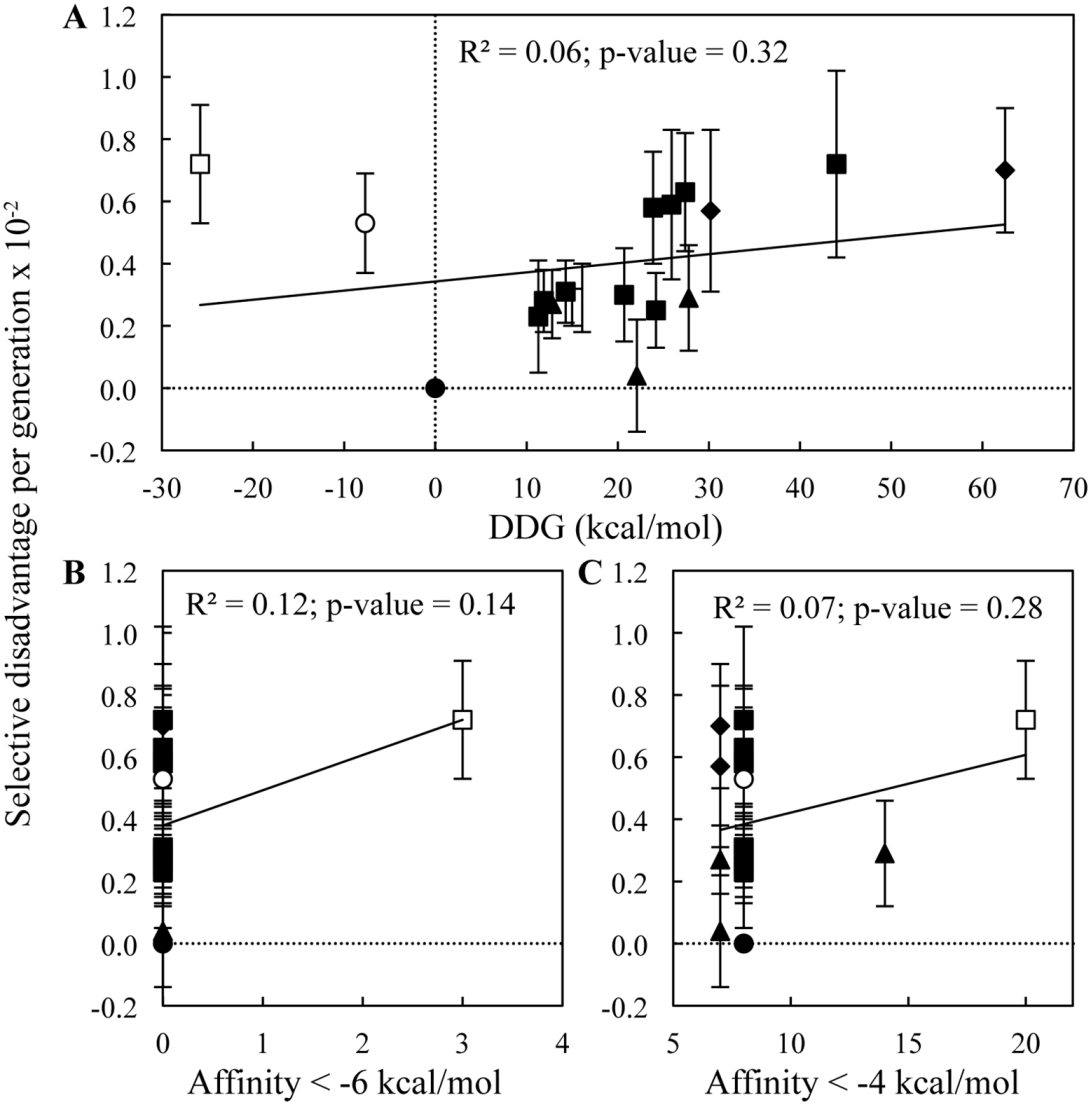
Selective disadvantage per generation of synonymous *tuf* alleles. Selective disadvantage as a function of **(A)** absolute changes in predicted mRNA free energy, and number of hexamers with a binding affinity to the anti-SD sequence in 16S rRNA of **(B)** < -6 kcal/mol and **(C)** < -4 kcal/mol. Values are shown for the *tufA* gene (filled circle) and synonymous *tuf* alleles with changes affecting codons for leucine (filled squares), proline (filled triangles), arginine (open squares), valine (open circles) and combinations of leucine and proline (filled diamonds). Results are shown as means ± 95% confidence interval. Black line indicates regression fit.

Previous studies that have investigated the effects of synonymous codons have found a correlation between the selective disadvantage of non-optimal synonymous codons and the change in predicted free energy for the synonymous alleles [11]. No such correlation was observed for the eighteen different synonymous *tuf* alleles (r-squared = 0.06; p-value 0.32) (Fig 3 and Table 1) suggesting that changes in mRNA stability are not responsible for the observed fitness cost.

**Fig 4:**
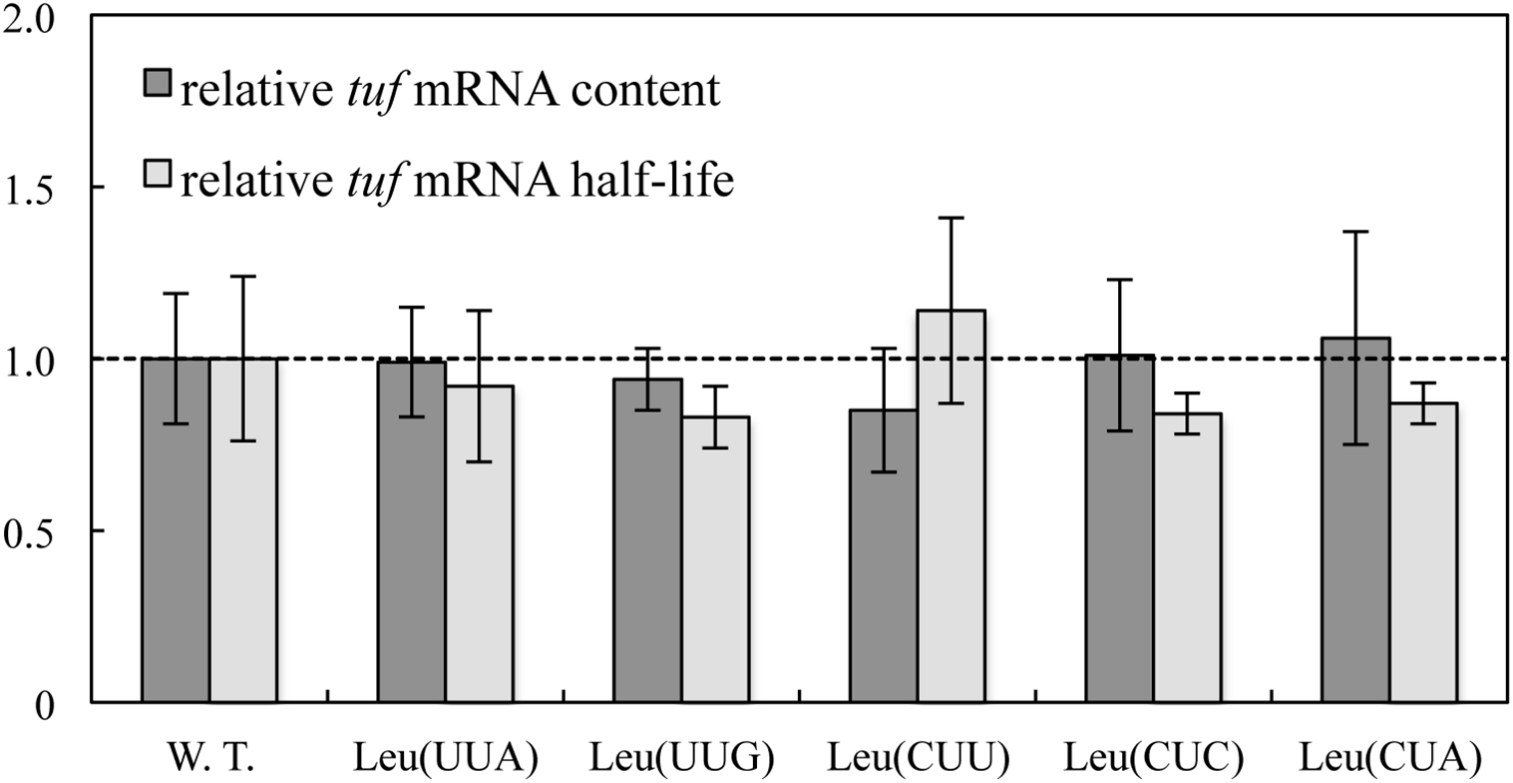
Analysis of *tuf* mRNA. Relative *tuf* mRNA content (dark grey) and relative *tuf* mRNA half-life (light grey) for wild-type *tufA* and synonymous *tuf* alleles with all leucine codons changed to UUA, UUG, CUU, CUC or CUA, respectively. Results are shown as means ± standard deviation.

A recent study showed that codon usage modulates both translation efficiency and mRNA stability [20]. Total *tuf* mRNA levels and mRNA half-lives were measured for the *tufA* allele and for the five *tuf* alleles with synonymous substitutions in all leucine codons, to investigate the effects of synonymous substitutions on the mRNA stability. There was no significant difference between the total mRNA level or the mRNA half-life for the *tufA* genes and any of the five synonymous *tuf* alleles (Fig 4), further confirming that the mRNA stability is not significantly affected by the synonymous substitutions.

Another study observed a correlation between the selective disadvantage of synonymous codons and the number hexamers with a strong binding affinity to the anti-SD sequence in 16S rRNA found within mRNA sequences [13]. The presence of these sequences might stall translating ribosomes and cause a decreased elongation speed. We found no significant correlation between the relative fitness values of the synonymous *tuf* alleles and the presence of hexamer sequences with a binding affinity of < -6 kcal/mol (r-squared = 0.12; p-value 0.14) or < -4 kcal/mol (r-squared = 0.07; p-value 0.28) (Fig 3 and Table 1).

**Fig 5:**
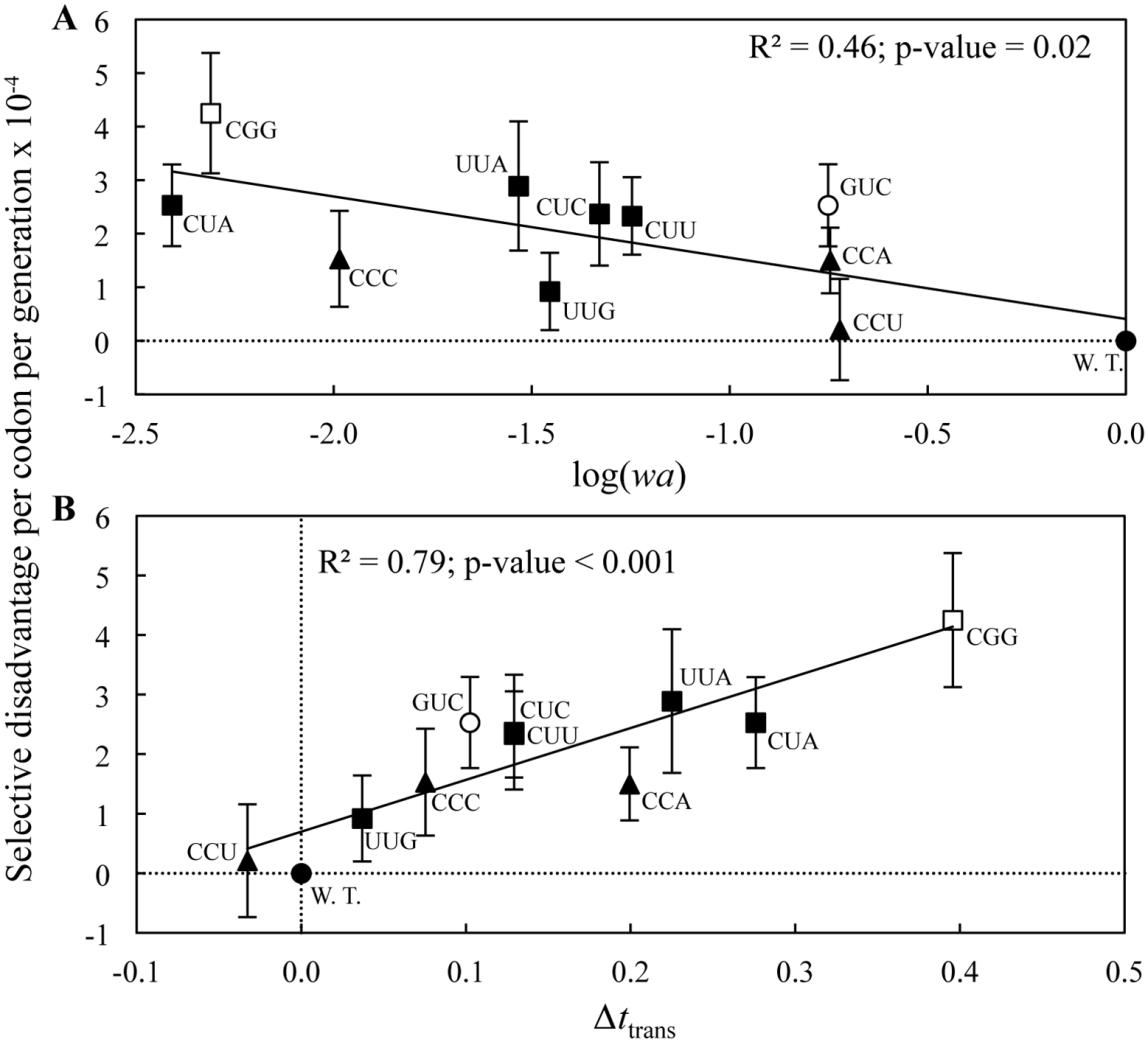
Selective disadvantage per codon per generation of synonymous *tuf* alleles. Selective disadvantage as a function of **(A)** logarithm of the relative adaptiveness and **(B)** increase in translational time for the *tufA* gene (filled circle) and synonymous *tuf* alleles with changes affecting codons for leucine (filled squares), proline (filled triangles), arginine (open squares), valine (open circles) and combinations of leucine and proline (filled diamonds). Results are shown as means ± 95% confidence interval. Codons are indicated next to the marker. Black line indicates regression fit.

Having tested these factors that could potentially cause a selective disadvantage independent from codon usage we next asked whether the relative fitness cost of each synonymous codon change correlated with actual codon usage bias. The frequency with which a codon is used over synonymous codons can be expressed as the relative adaptiveness *wa* and the logarithm of the relative adaptiveness is expected to be a linear function related to selection [21]. The log(*wa*) values, calculated using a set of highly expressed genes in *Salmonella* (Materials and Methods), were then correlated with the experimentally measured fitness costs. There was a significant correlation such that the lower the log(*wa*) value of a codon the higher the fitness cost it caused when used in the *tuf* genes (r-squared = 0.46; p-value 0.02) (Fig 5A and Table 1). We conclude that the fitness costs associated with the novel *tuf* alleles are a result of the switch from optimal codon usage to sub-optimal synonymous codon usage, and that the magnitude of the cost depends on the particular synonymous change.

We next addressed whether codon usage bias was associated with selection for translational speed or translational accuracy [6–8]. The frequency of codon usage has been shown to correlate to the concentration of cognate tRNA [22]. This correlation might be caused by a selection for translational speed, translational accuracy, or a combination of both. The speed with which a codon is decoded depends on the cellular concentration of the cognate tRNA [22, 23]. Therefore, a correlation between the cognate tRNA abundance for a particular a codon and its selective disadvantage is expected if translational speed is a selective force. This correlation is not necessarily exclusive to the selection for translational speed. The accuracy with which a codon is translated is also dependent on the abundance of cognate and near-cognate tRNA species [24]. One major difference between these two selective forces is in the predicted effects that the locations of synonymous codons will have on fitness. If translational speed were the major selective force on codon usage bias then it is expected that the observed fitness cost of sub-optimal codon usage should be proportional to the resulting increase in translation time. In such a case the degree of selective disadvantage should be independent of the positions of the synonymous mutations in the coding sequence and for each synonymous codon there should be a correlation between the number of substitutions and the resulting selective disadvantage. In contrast, if translational accuracy were the major selective force driving codon usage bias, the fitness cost of sub-optimal codon usage should be highly position dependent and no correlation should be observed between the number of substitutions and the selective disadvantage. This is because missense errors will have different magnitudes of effect on protein function dependent on where in the protein they are located. The eighteen synonymous *tuf* alleles tested here have synonymous mutations that are located in four entirely independent sets of positions in the *tuf* gene. Analysis of the data shows that the magnitude of the selective disadvantage of these non-optimal codons is strongly correlated with the relative change in translational time for the synonymous codons compared to the optimal codon (Fig 5B). This correlation is valid for all eighteen synonymous *tuf* alleles and is independent of the positions of the synonymous changes (r-squared = 0.79; p-value < 0.001) (Table 1 and Fig 5B). Additionally, a strong correlation between the number of synonymous substitutions and the selective disadvantage was observed for three synonymous leucine alleles (Fig 2). These correlations support the hypothesis that translational speed, rather than translational accuracy, is the major force selecting for codon usage bias, at least in the highly expressed *tuf* genes.

## Discussion

The relative usage of synonymous codons within the genomes of free-living organisms is not random but is subject to a strong bias. The strength of this bias varies between genes within the same genome (highly expressed genes show the greatest bias) but it can also vary according to position within individual genes. The common factor linking these phenomena is a selection to optimize the translation of mRNAs. Thus, the 5’ ends of highly expressed genes are optimized for translational initiation. This optimization is accomplished by a selection for reduced mRNA secondary structure around the Shine-Dalgarno site [10] and the existence of a ramp of rare, slowly translated codons early in the coding sequence, that help to ensure an even spacing of ribosomes on the mRNA, to reduce ribosomal traffic jams during further elongation [25]. Codon usage throughout the remainder of a highly expressed gene is selected to optimize translational speed, accuracy and co-translational protein folding. Optimal codons are translated at a higher speed [26] and more accurately [6, 27], while the presence of Shine-Dalgarno-like sequences, rare codons and secondary mRNA structures can be selected to locally slow down elongation and thereby support correct protein folding [25, 28, 29].

We set out to measure the magnitude of selection for optimal codons in highly expressed bacterial genes and asked whether the observed synonymous codon usage bias is selected to maximize translation speed or translation accuracy. The synonymous *tuf* alleles synthetized for use in this study were designed to have no changes in the first forty codons so as to remove any effects from changes in translational initiation on the results. It has previously been shown that some rare codons in highly expressed genes can be required for proper protein production [13], therefore in this study only the most common codons in *tuf* were changed to reduce the possible impact of site-specific codon selection. All eighteen synonymous *tuf* alleles assayed caused a measureable selective disadvantage within a small range from 0.2–4.2 x 10^−4^ per codon per generation. For three synonymous leucine alleles it was shown that the selective disadvantage is proportional to the number of synonymous substitutions, indicating that all synonymous substitutions contribute approximately equally to the total selective disadvantage. In two cases where codons for two different amino acids were simultaneously changed the magnitude of the effect was consistent with being the sum of the effects of changing the individual amino acids. The fact that no outliers were observed, that there was no change in *tuf* mRNA content or half-life, and that the observed fitness costs do not correlate with the predicted mRNA folding energies, or with the presence of sequences with strong binding affinity to anti-SD sequences in 16S rRNA, supports the hypothesis that the observed selective disadvantages of the synonymous *tuf* alleles are caused by a general mechanism rather than by specific effects caused by local changes. This conclusion is further supported by the correlation observed between the relative adaptiveness of the synonymous codons and their respective selective disadvantage. We therefore conclude that the observed fitness costs represents the magnitude of selection for optimal codons in highly expressed genes.

A previous study has investigated the fitness effects of synonymous substitutions in the bacterial virus T7 [30]. The average selective disadvantage per single nucleotide change was determined to be 4 x 10^−2^ doublings per hour. A selective disadvantage of 2 x 10^−4^ per codon per generation in *Salmonella*, as measured in our study, equals approximately 6 x 10^−4^ doublings per hour. This shows that the average selective disadvantage per synonymous substitution is almost 100 fold smaller in *Salmonella* than in T7. This difference is most likely a reflection of the differences in growth rate between the two species (3 doublings per hour for *Salmonella* and 43 doublings per hour for T7).

There is an ongoing discussion concerning whether translational speed or translational accuracy is the major selective force shaping codon bias in highly expressed genes. A recent study showed in a *Neurospora* cell-free translation system that commonly used codons are translated with a higher velocity than uncommon ones [31]. This is in agreement with our results that the observed fitness costs strongly correlate with the increase in translation time for synonymous codons compared to the optimal codon. This finding strongly supports the idea that translational speed is the major selective force for the observed codon usage bias. The calculation of the translation time is solely based on tRNA abundance data. Recent data show that the accuracy of wobble discrimination of tRNAs can vary within an order of magnitude [32] which will effect the actual translation time of respective codons. This variation has not been determined for any of the tRNAs that are part of this study and are therefore not part of the predicted increase in translation time. This neglected variation might explain the remaining scatter in the correlation plot (Fig 5B).

Since translational accuracy is also affected by tRNA isoacceptor abundance it is not possible to definitively rule out that this observed correlation between codon usage and relative fitness might also reflect an affect on translational accuracy. It is interesting though that the observed correlation held true even when codons were changed at independent positions and that there was a correlation between the number of synonymous substitutions of a particular codon and the selective disadvantage. This was expected if the selective disadvantage of changing synonymous codons were caused by selection for translational speed but not necessarily if it were caused by selection for translational accuracy. We therefore conclude that selection for translational speed is the most likely force that selects for codon bias in the codons of the *tuf* gene that were investigated in this study.

Our results are in agreement with a previously suggested model on the effects of codon usage bias on bacterial translation and growth rate [1, 10, 33]. The growth rate of unicellular organisms, such as *Salmonella*, is under optimal growth conditions directly proportional to the rate of protein production. The initiation of translation is the rate-limiting step in protein production and a decrease in translational elongation speed does not have a direct effect on the rate of production of the protein in question. Nevertheless, a reduction in translational elongation speed will cause ribosomes to stay longer on the mRNA and this has the effect of decreasing the cellular pool of free ribosomes. This decrease in the pool of free ribosomes will cause a decrease in the overall translational initiation rate in the cell and thereby decrease the overall rate of protein production in the cell. Selection-mutation-drift theory has been used to calculate the selective disadvantage *s* caused by a reduced translational elongation speed due to the presence of a rare codon [1]. The fitness cost of a single codon change from a frequent to a rare codon outside of the initiation region of mRNA is estimated to be *s* = 0.01*p*, where *p* is the relative abundance of the protein in question [1]. The relative abundance of EF-Tu at 9% of the total protein mass [19] which, using the model of Bulmer, would lead to a predicted selective disadvantage of *s* = 9 x 10^−4^ per codon per generation for synonymous codon changes in the *tuf* genes. This theoretical value agrees well with the range of values measured experimentally in this study (0.2–4 x 10^−4^ per codon per generation).

There are several reasons why a quantitative measure of the selective value of synonymous codon usage bias could be important and informative. In both synthetic biology and in biotechnology the aim is to express heterologous genetic information efficiently [34], while minimizing the risk of selecting and accumulating mutant variants or causing excessive disruption to cellular physiology. This is usually achieved by creating heterologous genes that carry the optimal codons for highly expressed genes in the host organism [35]. However, there are several specific problems that could arise, and that would necessitate the use of synonymous codons at particular sites. Because our measurements show that the selective value of alternative synonymous codons differs significantly the particular synonymous codon introduced could be chosen rationally. These situations include for example: (i) avoiding the creation of known RNase cleavage sites; (ii) avoiding sequence contexts associated with frameshifting or other translational errors; (iii) optimizing protein folding kinetics, a process that could be enhanced by translational pauses at particular positions [36]; and (iv) reducing excessive demand on particular aa-tRNA species, by spreading demand over alternative aa-tRNA's that are almost equally well translated. Having access to quantitative values for alternative synonymous codons would facilitate a rational choice of codon replacement in synthetic biology and biotechnology applications to maximize gene expression while minimizing the potential for mutation or physiological disruption.

This study provides the first experimental measurement of the strength of selection that acts on synonymous codon usage bias in highly expressed genes, shows that it varies between different codons, and suggests that it is selected to optimize translational speed in highly expressed genes in bacteria.

## Materials and Methods

### Construction of synonymous *tuf* alleles

The design of synonymous *tuf* alleles was based on the *Salmonella* enterica serovar Typhimurium LT2 *tufA* sequence. The frequency of all codons in the *tufA* sequence was calculated, excluding the first forty codons to reduce the impact N-terminal codon usage bias (S2 Table) [25, 37]. The particular amino acids included in this study were chosen based on the following criteria: (i) that multiple synonymous codons and tRNA isoacceptors exist, (ii) that the amino acid is frequently found in the EF-Tu sequence and (iii) that the codon usage of the respective amino acid is highly biased within the *tufA* sequence. The amino acids leucine and proline matched each of these requirements very well and were chosen for the design of synonymous *tuf* alleles. Five *tuf* alleles with synonymous leucine codon usage were synthesized. In each of these alleles all leucine codons were changed to UUA, UUG, CUU, CUC or CUA, respectively. Additionally, six *tuf* alleles were synthesized in which the leucine codons in only the first or the second half of the *tuf* gene were changed to UUA, CUC or CUA. Three *tuf* alleles with synonymous proline codons were synthesized where all proline codons were changed to CCU, CCC or CCA. Two more *tuf* alleles were synthesized to examine the effect of changing the codon bias of more than one amino acid at the time. In the first allele all leucine codons were changed to UUG and all proline codons to CCU. The second allele uses only the leucine codon UUA and the proline codon CCA. Arginine and valine were chosen for two additional synonymous tuf alleles to include more amino acids in this study. For both of these amino acids (arginine and valine) several codons in *tufA* are not encoded by the most frequently used codon. Only the codons that are encoded by the most frequently used codon were changed to simplify the analysis. These two synonymous *tuf* alleles were synthesized with all arginine CGU codons changed to CGG or all valine GUU codons to GUC, respectively. In total, eighteen distinct synonymous *tuf* alleles were designed (S1 Table) and were synthesized by Life Technologies (Darmstadt, Germany). Isogenic strains that carry the altered *tuf* alleles on the chromosome were constructed. The synthesized alleles were amplified by PCR and inserted into the chromosome replacing both of the genes, *tufA* and *tufB*, by recombineering [38] and sucrose counter selection [39]. Each synonymous *tuf* allele supported viability when present as the only *tuf* gene in the chromosome, regardless of whether it was placed at the *tufA* or *tufB* location. All strains used in fitness measurements carried a synonymous *tuf* allele at both *tuf* locations.

### Bacterial strains and growth conditions

All strains are derived from *S*. Typhimurium LT2. Bacteria were grown at 37°C in Luria-Bertani broth (LB) and on plates of LB medium solidified with 1.5% agar (Oxoid) (LA-plates). Sucrose counter selection was performed on salt-free LA-plates containing 5% sucrose.

### Fitness measurements

Relative bacterial fitness was determined by strain competition. Competitor strains were marked by insertion of a chromosomal YFP or BFP cassette. Strains that carry the synonymous *tuf* alleles in both *tuf* loci were compared to a strain that carries the *tufA* gene in both *tuf* loci. Briefly, twelve independent cultures of each of the twelve strains with a synonymous *tuf* allele and YFP marker, and twelve independent cultures of the strain with two *tufA* genes and YFP marker, were competed against a single reference culture with BFP marker. The ratio of the two competing strains was determined every ten generations using a flow cytometer to determine the selection coefficient for each of the independent competitions. The selective disadvantage of the synonymous *tuf* allele was defined as the average difference between the selection coefficient of the strains with the synonymous *tuf* alleles and the strains with the *tufA* genes. Each competition was then repeated as described above but with swapped dyes to account for potential influences of the dyes on the competition.

Competitions were performed in 2 mL LB and started with approximately 10^6^ cfu/mL of each competitor. Cultures were grown at 37°C and 1,000-fold diluted after every overnight of growth (10 generations of growth per day). Competitions were performed for 30 generations and the ratio of competitors was determined every 10 generations using a flow cytometer. Selection coefficients were calculated as previously described [40]. A collection of 168 competitions of a strain with two *tufA* genes against the reference strains shows that the selection coefficients within a range of 0.8% per generation of the median follow a normal distribution (Shapiro-Wilkins test; W = 0.991; p-value = 0.58) with outliers outside this region (S2 Fig). Isolation and competition of some of these outliers confirmed that outlier strains had a selection coefficient that differed significantly from that of the original strain suggesting a genetic change being responsible. Outliers were removed from the analysis to increase the accuracy of the fitness measurements since these outliers do not represent the actual fitness of the original strain.

The twelve independent selection coefficients that were determined for every strain were compared to each other to account for outliers within the measurements. Every measurement that differed by more that 0.8% per generation from the median within the group was removed from further analysis. All data were normalized to the average selection coefficient of the *tufA* strain in the respective competition to be able to compare results from the dye swaps. The normalized selection coefficients from the 24 independent competitions with the synonymous *tuf* allele were grouped into one group and the normalized selection coefficients from the 24 independent competitions of the strain with two *tufA* genes were grouped into a second group. A two-tailed t-test was performed to compare the two groups. The selective disadvantage of the synonymous *tuf* allele was then defined as the average difference between the two groups.

### Calculations of selective disadvantage per codon

The relative fitness *ω* of a strain with a single mutation is given as 1-*s*, where *s* is the selective disadvantage of the mutation. The relative fitness of a strain with multiple mutations is given as

$$\omega =\prod_{i=1}^{N}\left(1-s_{i}\right)$$ (1)

where N is the total number of mutations and *s*_i_ is the selective disadvantage of the mutation i. If all mutations have the same selective disadvantage, then

$$\omega ={\left(1-s\right)}^{N}$$ (2)

so that the selective disadvantage per codon can be calculated by

$$s=1-\sqrt[{N}]{\omega }.$$ (3)

### EF-Tu mRNA analysis

Total *tuf* mRNA content and *tuf* mRNA half-lives were determined by a rifampicin run-out experiment. Bacterial cultures were grown in LB at 37°C until the OD_600nm_ was 0.2 to 0.3. At this point, a time zero sample was taken and rifampicin was added to a final concentration of 200 mg L^-1^. Samples were taken at after 4, 8 and 12 minutes. Total RNA was extracted using RNeasy Mini Kit (Qiagen). Chromosomal DNA was removed by using DNase Turbo DNA-free (Ambion) and RNA was converted into cDNA by using high-capacity reverse transcription kit (Applied Biosystems). Total cDNA was diluted to 5 ng μL^-1^ with ddH_2_O. For each real-time PCR reaction, 5 μL of cDNA (undiluted and diluted 1:10 and 1:100), 12.5 μL SYBR green (Applied Biosystems), 1.25 μL of 6 μM forward and reverse primers and 5 μL ddH_2_O were mixed. The Eco RT-PCR system (Illumina) was used for running the PCR program and analyze the data. The thermal steps used were 10 min at 95°C followed by 40 cycles of 15 s at 95°C and 1 min at 60°C. Total *tuf* mRNA levels were determined using the time point zero samples and the following oligonucleotides: *tuf* gene, 5’-GAACGTACAAAACCGCAC and 5’-CGCCGTAGGTTTTAGCCA; *cysG* (reference gene 1), 5’-AAAAAGGTAAACGCGTGG and 5’- GGCACTACCGAGAAAGGAA; *hcaT* (reference gene 2), 5’-GCCGTGGCTGATTGTGATA and 5’-CCTGCAAACGAATCACCT; *idnT* (reference gene 3), 5’-TCCGGCGTTAATGGTACT and 5’-CCCCGACGCTAAGATTAAA. EF-Tu mRNA half-lives were determined using time points 0, 4, 8 and 12 minutes. Oligonucleotides for *tuf* mRNA were used as described above but *tmRNA*, a mRNA with long half-life, was used as a reference: *tmRNA* (reference gene 4), 5′-GGCGGTTGGCCTCGTAA and 5′-GTTATTAAGCTGCTAAAGCGTAGGTTT.

### Calculation of relative adaptiveness

Elongation factors (EF-Tu and EF-G) and ribosomal proteins (S1-S21 and L1-L36) were used as a set of highly expressed genes. The first forty codons of each gene were removed to reduce the influence of N-terminal codon bias. Relative synonymous codon usage (RSCU) was calculated as previously described [21]. The relative adaptiveness *wa* [21] was calculated by the formula

$$wa=RSCU_{syn}/RSCU_{wt}$$ (4)

### Calculation of increase in translational time

Experimental data on tRNA abundance *n* in *E*. *coli* K12 with a growth rate of 2.5 doublings per hour [22] were used to estimate the tRNA abundance in *Salmonella*. This should give a reasonable estimate since tRNA concentrations are correlated to tRNA copy numbers [22] and *E*. *coli* K12 and *Salmonella* LT2 have identical copy numbers for all but one of the relevant tRNA genes (S3 Table). The increase in translation time *Δt*_*trans*_ is

$$\Delta t_{trans}=1/n_{syn}-1/n_{wt}$$ (5)

where *n*_syn_ is the abundance of tRNA species that recognize the codons used in the synonymous *tuf* allele and *n*_wt_ is the abundance of tRNA species that recognize the codons used in the wild type *tuf* allele.

### Statistical analysis

Regression analysis to test correlations was performed using R, version 2.15.2.

## Supporting Information

S1 Fig Selective disadvantages of synonymous *tuf* alleles. Boxplots of selective disadvantages for *tufA* and synonymous *tuf* alleles. Whiskers indicate interquartile ranges.
(TIF)

S2 Fig Distribution of selection coefficients in competition experiments. Histogram of the selection coefficients from 168 measurements of a strain with a *tufA* gene in both *tuf* loci. Measurements that follow a normal distribution are shown in red and outliers in blue.
(TIF)

S1 Table Codon usage of relevant codons in synonymous *tuf* alleles^a^. ^a^ The first forty codons were excluded to reduce the impact N-terminal codon bias. All differences between the synonymous *tuf* alleles and *tufA* are shown in bold.
(DOCX)

S2 Table Codon usage in the *tufA* gene^a^. ^a^ The first forty codons were excluded to reduce the impact N-terminal codon bias.
(DOCX)

S3 Table Overview over tRNA dosage^a^. ^a^ Intracellular concentration of tRNA (μM) in ***E*. *coli*** K12 with a growth rate of 2.5 doublings per hour [22].
(DOCX)

## References

[1] Bulmer M. The selection-mutation-drift theory of synonymous codon usage. Genetics. 1991;129(3):897–907. Epub 1991/11/01. 1752426 10.1093/genetics/129.3.897 PMC1204756

[2] Kliman RM, Hey J. The effects of mutation and natural selection on codon bias in the genes of Drosophila. Genetics. 1994;137(4):1049–56. 7982559 10.1093/genetics/137.4.1049 PMC1206052

[3] Rocha EP. Codon usage bias from tRNA's point of view: redundancy, specialization, and efficient decoding for translation optimization. Genome Res. 2004;14(11):2279–86. 15479947 10.1101/gr.2896904 PMC525687

[4] Sharp PM, Cowe E, Higgins DG, Shields DC, Wolfe KH, Wright F. Codon usage patterns in Escherichia coli, Bacillus subtilis, Saccharomyces cerevisiae, Schizosaccharomyces pombe, Drosophila melanogaster and Homo sapiens; a review of the considerable within-species diversity. Nucleic acids research. 1988;16(17):8207–11. 3138659 10.1093/nar/16.17.8207 PMC338553

[5] Sharp PM, Bailes E, Grocock RJ, Peden JF, Sockett RE. Variation in the strength of selected codon usage bias among bacteria. Nucleic acids research. 2005;33(4):1141–53. Epub 2005/02/25. 15728743 10.1093/nar/gki242 PMC549432

[6] Stoletzki N, Eyre-Walker A. Synonymous codon usage in Escherichia coli: selection for translational accuracy. Mol Biol Evol. 2007;24(2):374–81. 17101719 10.1093/molbev/msl166

[7] Ikemura T. Correlation between the abundance of Escherichia coli transfer RNAs and the occurrence of the respective codons in its protein genes: a proposal for a synonymous codon choice that is optimal for the E. coli translational system. Journal of molecular biology. 1981;151(3):389–409. 6175758 10.1016/0022-2836(81)90003-6

[8] Akashi H. Synonymous codon usage in Drosophila melanogaster: natural selection and translational accuracy. Genetics. 1994;136(3):927–35. 8005445 10.1093/genetics/136.3.927 PMC1205897

[9] Hartl DL, Moriyama EN, Sawyer SA. Selection intensity for codon bias. Genetics. 1994;138(1):227–34. Epub 1994/09/01. 8001789 10.1093/genetics/138.1.227 PMC1206133

[10] Kudla G, Murray AW, Tollervey D, Plotkin JB. Coding-sequence determinants of gene expression in Escherichia coli. Science (New York, NY. 2009;324(5924):255–8. 10.1126/science.1170160 PMC3902468 19359587

[11] Lind PA, Berg OG, Andersson DI. Mutational robustness of ribosomal protein genes. Science (New York, NY. 2010;330(6005):825–7. Epub 2010/11/06. 10.1126/science.1194617 21051637

[12] Lind PA, Andersson DI. Fitness costs of synonymous mutations in the rpsT gene can be compensated by restoring mRNA base pairing. PLoS One. 2013;8(5):e63373 10.1371/journal.pone.0063373 23691039 PMC3655191

[13] Agashe D, Martinez-Gomez NC, Drummond DA, Marx CJ. Good codons, bad transcript: large reductions in gene expression and fitness arising from synonymous mutations in a key enzyme. Mol Biol Evol. 2013;30(3):549–60. 10.1093/molbev/mss273 23223712 PMC3563975

[14] Spanjaard RA, van Duin J. Translation of the sequence AGG-AGG yields 50% ribosomal frameshift. Proceedings of the National Academy of Sciences of the United States of America. 1988;85(21):7967–71. 3186700 10.1073/pnas.85.21.7967 PMC282334

[15] Kramer EB, Farabaugh PJ. The frequency of translational misreading errors in E. coli is largely determined by tRNA competition. RNA (New York, NY. 2007;13(1):87–96. 10.1261/rna.294907 PMC1705757 17095544

[16] Sorensen MA, Kurland CG, Pedersen S. Codon usage determines translation rate in Escherichia coli. Journal of molecular biology. 1989;207(2):365–77. 2474074 10.1016/0022-2836(89)90260-x

[17] Gouy M, Gautier C. Codon usage in bacteria: correlation with gene expressivity. Nucleic acids research. 1982;10(22):7055–74. Epub 1982/11/25. 6760125 10.1093/nar/10.22.7055 PMC326988

[18] Goetz RM, Fuglsang A. Correlation of codon bias measures with mRNA levels: analysis of transcriptome data from Escherichia coli. Biochem Biophys Res Commun. 2005;327(1):4–7. 15629421 10.1016/j.bbrc.2004.11.134

[19] Tubulekas I, Hughes D. Growth and translation elongation rate are sensitive to the concentration of EF-Tu. Molecular microbiology. 1993a;8(4):761–70. 8332067 10.1111/j.1365-2958.1993.tb01619.x

[20] Boel G, Letso R, Neely H, Price WN, Wong KH, Su M, et al Codon influence on protein expression in E. coli correlates with mRNA levels. Nature. 2016;529(7586):358–63. 10.1038/nature16509 26760206 PMC5054687

[21] Sharp PM, Li WH. The codon Adaptation Index—a measure of directional synonymous codon usage bias, and its potential applications. Nucleic acids research. 1987;15(3):1281–95. Epub 1987/02/11. 3547335 10.1093/nar/15.3.1281 PMC340524

[22] Dong H, Nilsson L, Kurland CG. Co-variation of tRNA abundance and codon usage in Escherichia coli at different growth rates. Journal of molecular biology. 1996;260(5):649–63. 8709146 10.1006/jmbi.1996.0428

[23] Varenne S, Buc J, Lloubes R, Lazdunski C. Translation is a non-uniform process. Effect of tRNA availability on the rate of elongation of nascent polypeptide chains. Journal of molecular biology. 1984;180(3):549–76. 6084718 10.1016/0022-2836(84)90027-5

[24] Shah P, Gilchrist MA. Effect of Correlated tRNA Abundances on Translation Errors and Evolution of Codon Usage Bias. PLoS Genet. 2010;6(9):e1001128 10.1371/journal.pgen.1001128 20862306 PMC2940732

[25] Tuller T, Carmi A, Vestsigian K, Navon S, Dorfan Y, Zaborske J, et al An evolutionarily conserved mechanism for controlling the efficiency of protein translation. Cell. 2010;141(2):344–54. 10.1016/j.cell.2010.03.031 20403328

[26] Sorensen MA, Pedersen S. Absolute in vivo translation rates of individual codons in Escherichia coli. The two glutamic acid codons GAA and GAG are translated with a threefold difference in rate. Journal of molecular biology. 1991;222(2):265–80. 1960727 10.1016/0022-2836(91)90211-n

[27] Drummond DA, Wilke CO. Mistranslation-induced protein misfolding as a dominant constraint on coding-sequence evolution. Cell. 2008;134(2):341–52. 10.1016/j.cell.2008.05.042 18662548 PMC2696314

[28] Li GW, Oh E, Weissman JS. The anti-Shine-Dalgarno sequence drives translational pausing and codon choice in bacteria. Nature. 2012;484(7395):538–41. 10.1038/nature10965 22456704 PMC3338875

[29] Zhang G, Hubalewska M, Ignatova Z. Transient ribosomal attenuation coordinates protein synthesis and co-translational folding. Nat Struct Mol Biol. 2009;16(3):274–80. 10.1038/nsmb.1554 19198590

[30] Bull JJ, Molineux IJ, Wilke CO. Slow fitness recovery in a codon-modified viral genome. Mol Biol Evol. 2012;29(10):2997–3004. 22532576 10.1093/molbev/mss119 PMC3457771

[31] Yu CH, Dang Y, Zhou Z, Wu C, Zhao F, Sachs MS, et al Codon Usage Influences the Local Rate of Translation Elongation to Regulate Co-translational Protein Folding. Molecular cell. 2015;59(5):744–54. 10.1016/j.molcel.2015.07.018 26321254 PMC4561030

[32] Zhang J, Ieong KW, Johansson M, Ehrenberg M. Accuracy of initial codon selection by aminoacyl-tRNAs on the mRNA-programmed bacterial ribosome. Proceedings of the National Academy of Sciences of the United States of America. 2015;112(31):9602–7. 10.1073/pnas.1506823112 26195797 PMC4534242

[33] Andersson SG, Kurland CG. Codon preferences in free-living microorganisms. Microbiol Rev. 1990;54(2):198–210. Epub 1990/06/01. 2194095 10.1128/mr.54.2.198-210.1990 PMC372768

[34] Hatfield GW, Roth DA. Optimizing scaleup yield for protein production: Computationally Optimized DNA Assembly (CODA) and Translation Engineering. Biotechnology annual review. 2007;13:27–42. 17875472 10.1016/S1387-2656(07)13002-7

[35] Welch M, Govindarajan S, Ness JE, Villalobos A, Gurney A, Minshull J, et al Design parameters to control synthetic gene expression in Escherichia coli. PLoS One. 2009;4(9):e7002 10.1371/journal.pone.0007002 19759823 PMC2736378

[36] Pechmann S, Frydman J. Evolutionary conservation of codon optimality reveals hidden signatures of cotranslational folding. Nat Struct Mol Biol. 2013;20(2):237–43. 10.1038/nsmb.2466 23262490 PMC3565066

[37] Fredrick K, Ibba M. How the sequence of a gene can tune its translation. Cell. 2010;141(2):227–9. 10.1016/j.cell.2010.03.033 20403320 PMC2866089

[38] Datta S, Costantino N, Court DL. A set of recombineering plasmids for gram-negative bacteria. Gene. 2006;379:109–15. 16750601 10.1016/j.gene.2006.04.018

[39] Gay P, Le Coq D, Steinmetz M, Berkelman T, Kado CI. Positive selection procedure for entrapment of insertion sequence elements in gram-negative bacteria. Journal of bacteriology. 1985;164(2):918–21. 2997137 10.1128/jb.164.2.918-921.1985 PMC214340

[40] Dykhuizen DE. Experimental studies of natural selection in bacteria. Annual Review of Ecology and Systematics. 1990;21:373–98.

