# Supplementary figures and images for: The Selective Advantage of Synonymous Codon Usage Bias in Salmonella
Source: PLoS Genet. 2016 Mar 10;12(3):e1005926. doi: 10.1371/journal.pgen.1005926 (PMC4786093; doi:10.1371/journal.pgen.1005926)

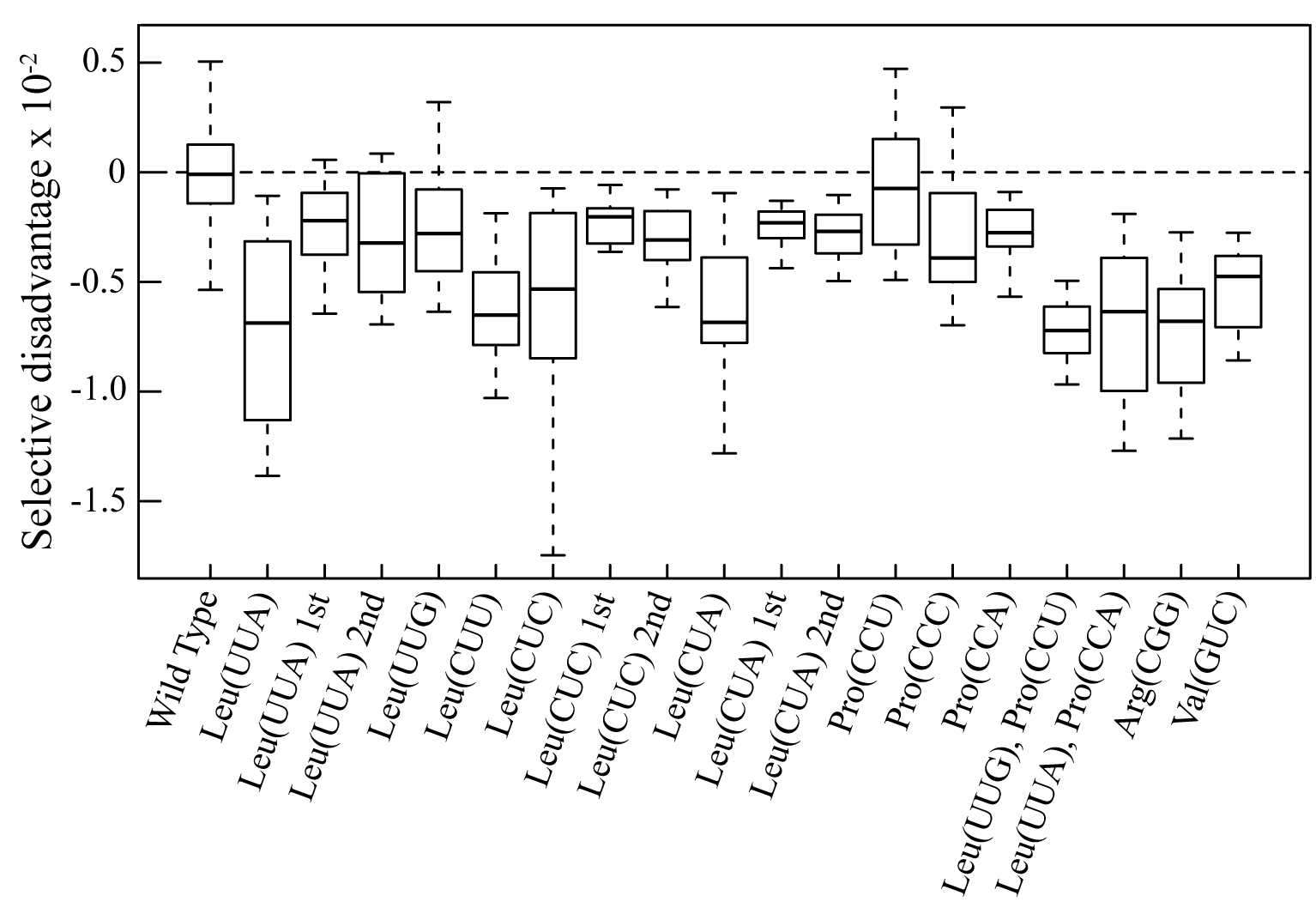

Supplement: S1 Fig — Boxplots of selective disadvantages for tufA and synonymous tuf alleles. Whiskers indicate interquartile ranges. (TIF) [file pgen.1005926.s001.tif]

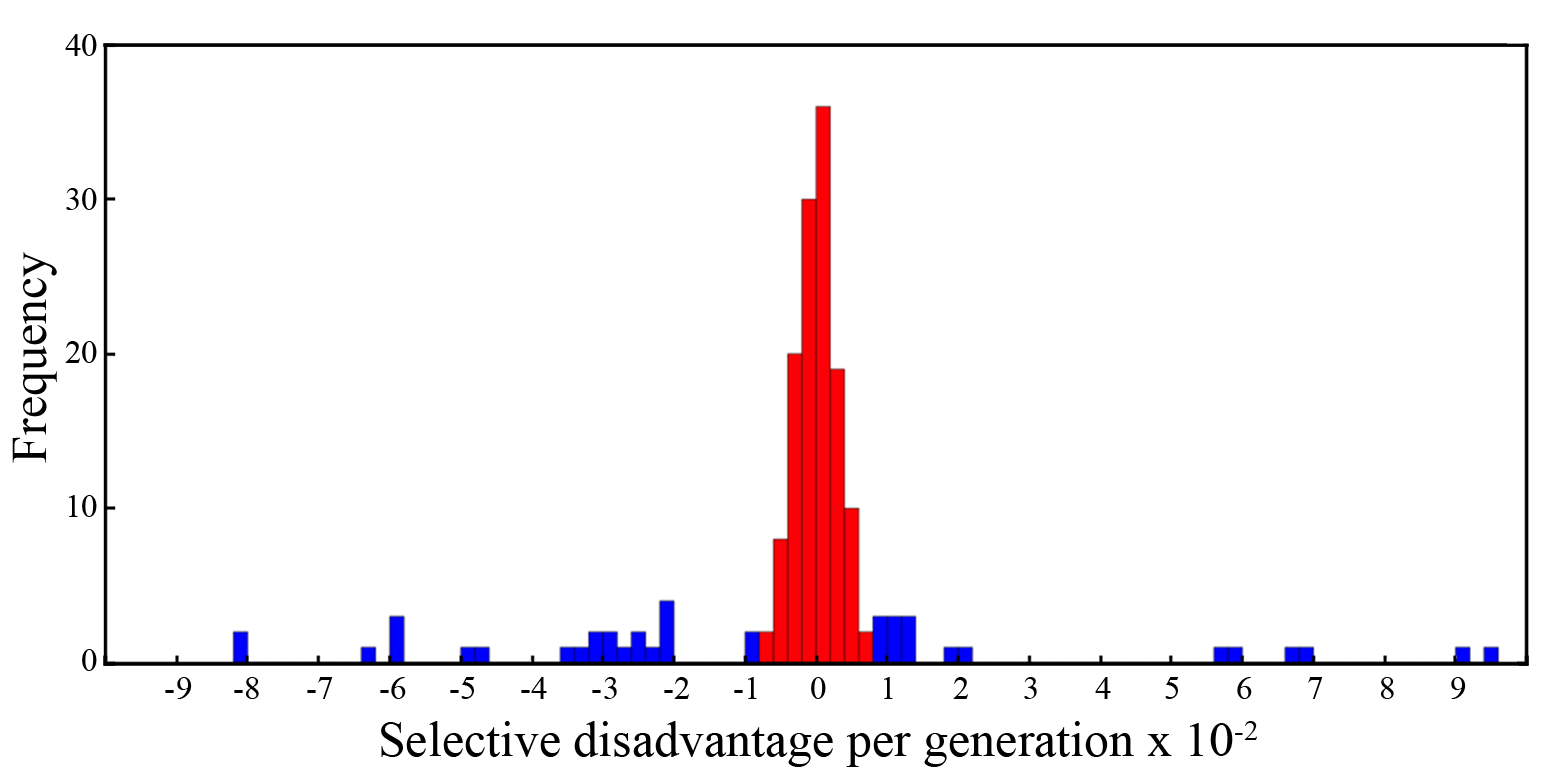

Supplement: S2 Fig — Histogram of the selection coefficients from 168 measurements of a strain with a tufA gene in both tuf loci. Measurements that follow a normal distribution are shown in red and outliers in blue. (TIF) [file pgen.1005926.s002.tif]
