# Supplementary material for: The Selective Advantage of Synonymous Codon Usage Bias in Salmonella
Source: PLoS Genet. 2016 Mar 10;12(3):e1005926. doi: 10.1371/journal.pgen.1005926 (PMC4786093; doi:10.1371/journal.pgen.1005926)
Supplement: S1 Table — a The first forty codons were excluded to reduce the impact N-terminal codon bias. All differences between the synonymous tuf alleles and tufA are shown in bold. (DOCX) [file pgen.1005926.s003.docx]

S1 Table. Codon usage of relevant codons in synonymous *tuf* alleles^a^.

| *tuf* allele | Leucine | | | | | |  | Proline | | | |  | Argenine | |  | Valine | |
| --- | --- | --- | --- | --- | --- | --- | --- | --- | --- | --- | --- | --- | --- | --- | --- | --- | --- |
|  | **UUA** | **UUG** | **CUU** | **CUC** | **CUA** | **CUG** |  | **CCU** | **CCC** | **CCA** | **CCG** |  | **CCG** | **CGU** |  | **GUC** | **GUU** |
| *tufA* | 0 | 0 | 0 | 0 | 0 | 25 |  | 0 | 0 | 1 | 18 |  | 0 | 17 |  | 0 | 21 |
| *tufLeu(UUA)* | **25** | 0 | 0 | 0 | 0 | **0** |  | 0 | 0 | 1 | 18 |  | 0 | 17 |  | 0 | 21 |
| *tufLeu(UUA) 1^st^ half* | **13** | 0 | 0 | 0 | 0 | **12** |  | 0 | 0 | 1 | 18 |  | 0 | 17 |  | 0 | 21 |
| *tufLeu(UUA) 2^nd^ half* | **12** | 0 | 0 | 0 | 0 | **13** |  | 0 | 0 | 1 | 18 |  | 0 | 17 |  | 0 | 21 |
| *tufLeu(UUG)* | 0 | **25** | 0 | 0 | 0 | **0** |  | 0 | 0 | 1 | 18 |  | 0 | 17 |  | 0 | 21 |
| *tufLeu(CUU)* | 0 | 0 | **25** | 0 | 0 | **0** |  | 0 | 0 | 1 | 18 |  | 0 | 17 |  | 0 | 21 |
| *tufLeu(CUC)* | 0 | 0 | 0 | **25** | 0 | **0** |  | 0 | 0 | 1 | 18 |  | 0 | 17 |  | 0 | 21 |
| *tufLeu(CUC) 1^st^ half* | 0 | 0 | 0 | **13** | 0 | **12** |  | 0 | 0 | 1 | 18 |  | 0 | 17 |  | 0 | 21 |
| *tufLeu(CUC) 2^nd^ half* | 0 | 0 | 0 | **12** | 0 | **13** |  | 0 | 0 | 1 | 18 |  | 0 | 17 |  | 0 | 21 |
| *tufLeu(CUA)* | 0 | 0 | 0 | 0 | **25** | **0** |  | 0 | 0 | 1 | 18 |  | 0 | 17 |  | 0 | 21 |
| *tufLeu(CUA) 1^st^ half* | 0 | 0 | 0 | 0 | **13** | **12** |  | 0 | 0 | 1 | 18 |  | 0 | 17 |  | 0 | 21 |
| *tufLeu(CUA) 2^nd^ half* | 0 | 0 | 0 | 0 | **12** | **13** |  | 0 | 0 | 1 | 18 |  | 0 | 17 |  | 0 | 21 |
| *tufPro(CCU)* | 0 | 0 | 0 | 0 | 0 | 25 |  | **19** | 0 | **0** | **0** |  | 0 | 17 |  | 0 | 21 |
| *tufPro(CCC)* | 0 | 0 | 0 | 0 | 0 | 25 |  | 0 | **19** | **0** | **0** |  | 0 | 17 |  | 0 | 21 |
| *tufPro(CCA)* | 0 | 0 | 0 | 0 | 0 | 25 |  | 0 | 0 | **19** | **0** |  | 0 | 17 |  | 0 | 21 |
| *tufLeu(UUG)Pro(CCU)* | 0 | **25** | 0 | 0 | 0 | **0** |  | **19** | 0 | **0** | **0** |  | 0 | 17 |  | 0 | 21 |
| *tufLeu(UUA)Pro(CCA)* | **25** | 0 | 0 | 0 | 0 | **0** |  | 0 | 0 | **19** | **0** |  | 0 | 17 |  | 0 | 21 |
| *tufArg(CCG)* | 0 | 0 | 0 | 0 | 0 | 25 |  | 0 | 0 | 1 | 18 |  | **17** | **0** |  | 0 | 21 |
| *tufVal(GUC)* | 0 | 0 | 0 | 0 | 0 | 25 |  | 0 | 0 | 1 | 18 |  | 0 | 17 |  | **21** | **0** |

^a^ The first forty codons were excluded to reduce the impact N-terminal codon bias. All differences between the synonymous *tuf* alleles and *tufA* are in bold.
