# Supplementary material for: The Selective Advantage of Synonymous Codon Usage Bias in Salmonella
Source: PLoS Genet. 2016 Mar 10;12(3):e1005926. doi: 10.1371/journal.pgen.1005926 (PMC4786093; doi:10.1371/journal.pgen.1005926)
Supplement: S2 Table — a The first forty codons were excluded to reduce the impact N-terminal codon bias. (DOCX) [file pgen.1005926.s004.docx]

S2 Table. Codon usage in the *tufA* gene^a^.

| Amino acid | Codon | N |  | Amino acid | Codon | N |  | Amino acid | Codon | N |  | Amino acid | Codon | N |
| --- | --- | --- | --- | --- | --- | --- | --- | --- | --- | --- | --- | --- | --- | --- |
| Phe | UUU | 0 |  | Ser | UCU | 6 |  | Tyr | UAU | 2 |  | Cys | UGU | 1 |
| Phe | UUC | 13 |  | Ser | UCC | 3 |  | Tyr | UAC | 7 |  | Cys | UGC | 2 |
| Leu | UUA | 0 |  | Ser | UCA | 0 |  | Ter | UAA | 1 |  | Ter | UGA | 0 |
| Leu | UUG | 0 |  | Ser | UCG | 0 |  | Ter | UAG | 0 |  | Trp | UGG | 1 |
|  |  |  |  |  |  |  |  |  |  |  |  |  |  |  |
| Leu | CUU | 0 |  | Pro | CCU | 0 |  | His | CAU | 1 |  | Arg | CGU | 17 |
| Leu | CUC | 0 |  | Pro | CCC | 0 |  | His | CAC | 7 |  | Arg | CGC | 5 |
| Leu | CUA | 0 |  | Pro | CCA | 1 |  | Gln | CAA | 0 |  | Arg | CGA | 0 |
| Leu | CUG | 25 |  | Pro | CCG | 18 |  | Gln | CAG | 8 |  | Arg | CGG | 0 |
|  |  |  |  |  |  |  |  |  |  |  |  |  |  |  |
| Ile | AUU | 3 |  | Thr | ACU | 9 |  | Asn | AAU | 0 |  | Ser | AGU | 0 |
| Ile | AUC | 25 |  | Thr | ACC | 13 |  | Asn | AAC | 6 |  | Ser | AGC | 0 |
| Ile | AUA | 0 |  | Thr | ACA | 0 |  | Lys | AAA | 13 |  | Arg | AGA | 0 |
| Met | AUG | 10 |  | Thr | ACG | 0 |  | Lys | AAG | 5 |  | Arg | AGG | 0 |
|  |  |  |  |  |  |  |  |  |  |  |  |  |  |  |
| Val | GUU | 21 |  | Ala | GCU | 9 |  | Asp | GAU | 5 |  | Gly | GGU | 16 |
| Val | GUC | 0 |  | Ala | GCC | 2 |  | Asp | GAC | 18 |  | Gly | GGC | 21 |
| Val | GUA | 8 |  | Ala | GCA | 4 |  | Glu | GAA | 26 |  | Gly | GGA | 0 |
| Val | GUG | 4 |  | Ala | GCG | 9 |  | Glu | GAG | 9 |  | Gly | GGG | 1 |

^a^ The first forty codons were excluded to reduce the impact N-terminal codon bias.
