# Supplementary material for: The Selective Advantage of Synonymous Codon Usage Bias in Salmonella
Source: PLoS Genet. 2016 Mar 10;12(3):e1005926. doi: 10.1371/journal.pgen.1005926 (PMC4786093; doi:10.1371/journal.pgen.1005926)
Supplement: S3 Table — a Intracellular concentration of tRNA (μM) in E. coli K12 with a growth rate of 2.5 doublings per hour [22]. (DOCX) [file pgen.1005926.s005.docx]

S3 Table. Overview of tRNA dosage in *Salmonella* LT2 and *E. coli* K12.

| Amino acid | Anticodon | N*_Salmonella_* | N*_E. coli_* | Concentration (µM)^a^ |
| --- | --- | --- | --- | --- |
| Arg | ACG | 4 | 4 | 25.57 |
| Arg | CCG | 1 | 1 | 2.30 |
| Leu | GAG | 1 | 1 | 5.93 |
| Leu | CAG | 4 | 4 | 22.20 |
| Leu | UAG | 1 | 1 | 3.17 |
| Leu | CAA | 1 | 1 | 9.30 |
| Leu | UAA | 1 | 1 | 3.78 |
| Pro | GGG | 1 | 1 | 3.75 |
| Pro | CGG | 1 | 1 | 2.67 |
| Pro | UGG | 1 | 1 | 2.56 |
| Val | GAC | 2 | 2 | 7.21 |
| Val | UAC | 4 | 5 | 20.39 |

^a^ Intracellular concentration of tRNA (µM) in *E. coli* K12 with a growth rate of 2.5 doublings per hour (1).

1. Dong H, Nilsson L, Kurland CG. Co-variation of tRNA abundance and codon usage in Escherichia coli at different growth rates. Journal of molecular biology. 1996;260(5):649-63.
